# Survival Motor Neuron Protein Regulates Stem Cell Division, Proliferation, and Differentiation in *Drosophila*

**DOI:** 10.1371/journal.pgen.1002030

**Published:** 2011-04-07

**Authors:** Stuart J. Grice, Ji-Long Liu

**Affiliations:** Medical Research Council Functional Genomics Unit, Department of Physiology, Anatomy, and Genetics, University of Oxford, Oxford, United Kingdom; University of California San Francisco, United States of America

## Abstract

Spinal muscular atrophy is a severe neurogenic disease that is caused by mutations in the human *survival motor neuron 1* (*SMN1*) gene. SMN protein is required for the assembly of small nuclear ribonucleoproteins and a dramatic reduction of the protein leads to cell death. It is currently unknown how the reduction of this ubiquitously essential protein can lead to tissue-specific abnormalities. In addition, it is still not known whether the disease is caused by developmental or degenerative defects. Using the *Drosophila* system, we show that SMN is enriched in postembryonic neuroblasts and forms a concentration gradient in the differentiating progeny. In addition to the developing *Drosophila* larval CNS, *Drosophila* larval and adult testes have a striking SMN gradient. When SMN is reduced in postembryonic neuroblasts using MARCM clonal analysis, cell proliferation and clone formation defects occur. These SMN mutant neuroblasts fail to correctly localise Miranda and have reduced levels of snRNAs. When SMN is removed, germline stem cells are lost more frequently. We also show that changes in SMN levels can disrupt the correct timing of cell differentiation. We conclude that highly regulated SMN levels are essential to drive timely cell proliferation and cell differentiation.

## Author Summary

Spinal muscular atrophy is a debilitating disease that affects the motor nervous system. The disease is caused by the reduction of the protein survival motor neuron (SMN), which is involved in the assembly of ubiquitous small nuclear ribonucleoproteins. As SMN is required in every cell, it is important to understand the differential functionality of the protein within developing tissues. In this paper, we identify stem cells as having the highest levels of SMN. The concentration of SMN then decreases in a declining gradient until it reaches its lowest level in differentiated cells. SMN reduction, using clonal analysis, slows stem cell division and can lead to stem cell loss. These defects correlate with a reduction in the U2 and U5 small nuclear RNAs and with the mislocalisation of Miranda protein in postembryonic neuroblasts. In addition, we show that the overexpression of SMN can change the timing of development and cell differentiation. This research highlights possible mechanisms explaining how SMN expression alterations may affect tissue development.

## Introduction

Proximal spinal muscular atrophy (SMA) is characterised by the loss of the α-motor neurons in the anterior horns of the spinal cord, leading to progressive paralysis, muscle wasting, and in the most severe cases, death. SMA, an autosomal recessive disease, is the most common genetic form of infant mortality with an incidence of 1 in 10,000 live births [1]. It is caused by mutations or deletions in the *survival motor neuron 1* (*SMN1*) gene which, together with a paralogue *SMN2*, lies within an inverted repeat on human chromosome 5q13 [2], [3]. Due to altered splicing efficiency *SMN2* produces levels of SMN protein that are too low to maintain healthy motor neurons [4], [5], [6].

SMN is a ubiquitously expressed protein and functions within a large multiprotein complex that recruits and assembles small nuclear ribonucleoproteins (snRNPs). snRNPs are components of the macromolecular spliceosome that catalyses the splicing of pre-mRNAs [7]. Additional functions that have also been attributed to SMN include the processing of additional RNA subclasses and mRNA processing and transport in axons [8], [9]. However, how the reduction of SMN protein leads to a neuronal specific disease remains elusive [10].

SMN protein is highly expressed in the early mouse, zebrafish and *Drosophila* embryos [11], [12], [13]. In whole mouse tissues, snRNP-associated SMN activity is down-regulated upon differentiation [11]. Developmental defects have been observed in a number of models, in particular zebrafish, which display early axonal branching defects [14]. However, it is still unknown which cell populations within the developing tissues have higher SMN levels and how the protein is regulated on an individual cell level.

To understand the role of SMN in disease it is therefore important to understand 1) the unique vulnerability of motor neurons to the deficiency of this ‘housekeeping gene’ 2) why a monogenic deficiency causes a wide spectrum of phenotypic severity and 3) whether defects in SMA are determined early in development or related to degeneration later in life [15].

This study uses the tractability of the *Drosophila* system to uncover how developing tissues respond to SMN level changes. Here we report observations of SMN expression in two well-defined tissues in *Drosophila*: the larval CNS, and the male germline. We found that in both tissues the stem cells display the highest levels of SMN. SMN levels then decrease in a consistent gradient as cells differentiate into mature neurons and sperm. If SMN is removed from stem cells, division is slower in the CNS and stem cell loss is more frequent in the testis. SMN mutant neuroblasts have abnormally localised Miranda, which is an adaptor protein that binds and facilitates the basal anchoring of *prospero* mRNA in neuroblasts. Proliferation defects also correlate with snRNP reduction in the developing CNS and in the germline. In the developing testis, we show that contraction of the SMN gradient leads to premature differentiation, while its expansion can repress differentiation. Taking these results together, we conclude that the tight regulation of SMN expression on a cellular level is important for stem cell division, proliferation and daughter cell differentiation.

## Results

### SMN reduction causes proliferation defects in larval CNS development

**Figure 1:**
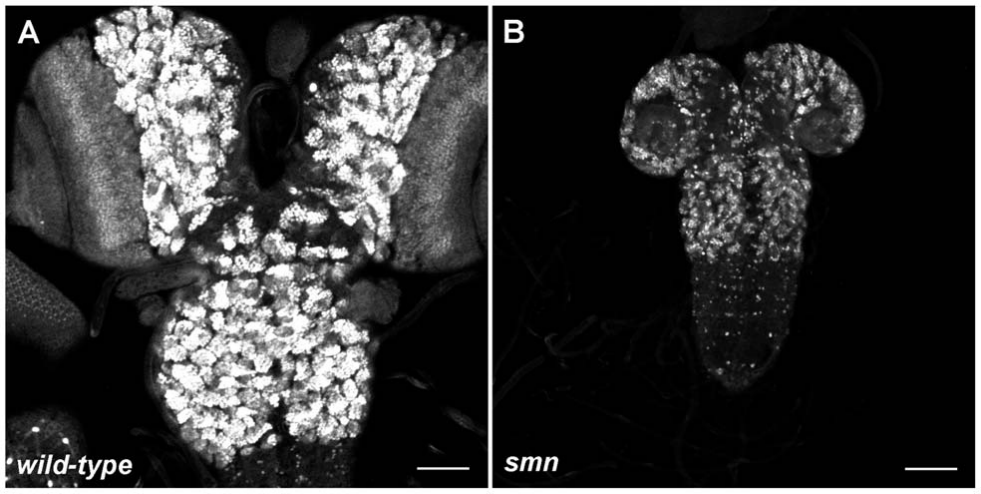
Growth defects in the SMN mutant CNS. Prospero staining in a wild-type (A) and *smn^B^* (B) mutant CNS at the same scale. Both CNS are taken from larvae with 3rd instar spiracles and mouth hooks (wild-type, 4.5 Days old; *smn^B^* 5.5 days old). The *smn^B^* CNS is considerably reduced in size and is comparable to that of a 2nd instar wild-type. Both the thoracic ganglion and the brain lobes have retarded growth. Scale bars, 50 µm.

We analysed the *Drosophila* loss of function alleles *smn^A^* (smn73Ao) and *smn^B^* which survive on maternally contributed wild-type SMN supplied from the heterozygous mother. *smn^A^* and *smn^B^* larvae develop motor defects and die at 2^nd^ and 3^rd^ instars, respectively [12], [16]. Prior to the onset of motor defects, both SMN mutants displayed CNS growth defects (Figure 1A, wild-type; 1B, less severe *smn^B^* only). As flies are holometabolic insects that undergo metamorphosis, their larval CNS comprises of regions of both fully differentiated and developing neurons for the respective larval and adult stages [17]. During larval life, postembryonic neuroblasts (pNBs) exit quiescence, enlarge and divide to generate the neurons, including motor neurons, required in the adult fly. These neurons remain in an immature state and can be observed in the brain lobes and the thoracic and abdominal ganglion. Both *smn^A^* and *smn^B^* mutant CNS were reduced in size when compared to wild-type at day 4 and 5. *smn^A^* CNS did not increase in size after this stage and the larvae die soon after the day 4 measurement. As Shpargel and colleagues previously described, *smn^B^* mutants can survive up to and beyond 8 days where they die as 3^rd^ instar larvae or as pseudopupae [16]. The size of *smn^B^* CNS at day 8 failed to reach that of CNS from wild-type larvae at day 5.

### SMN is up-regulated in pNBs and forms a concentration gradient that corresponds to the state of differentiation

**Figure 2:**
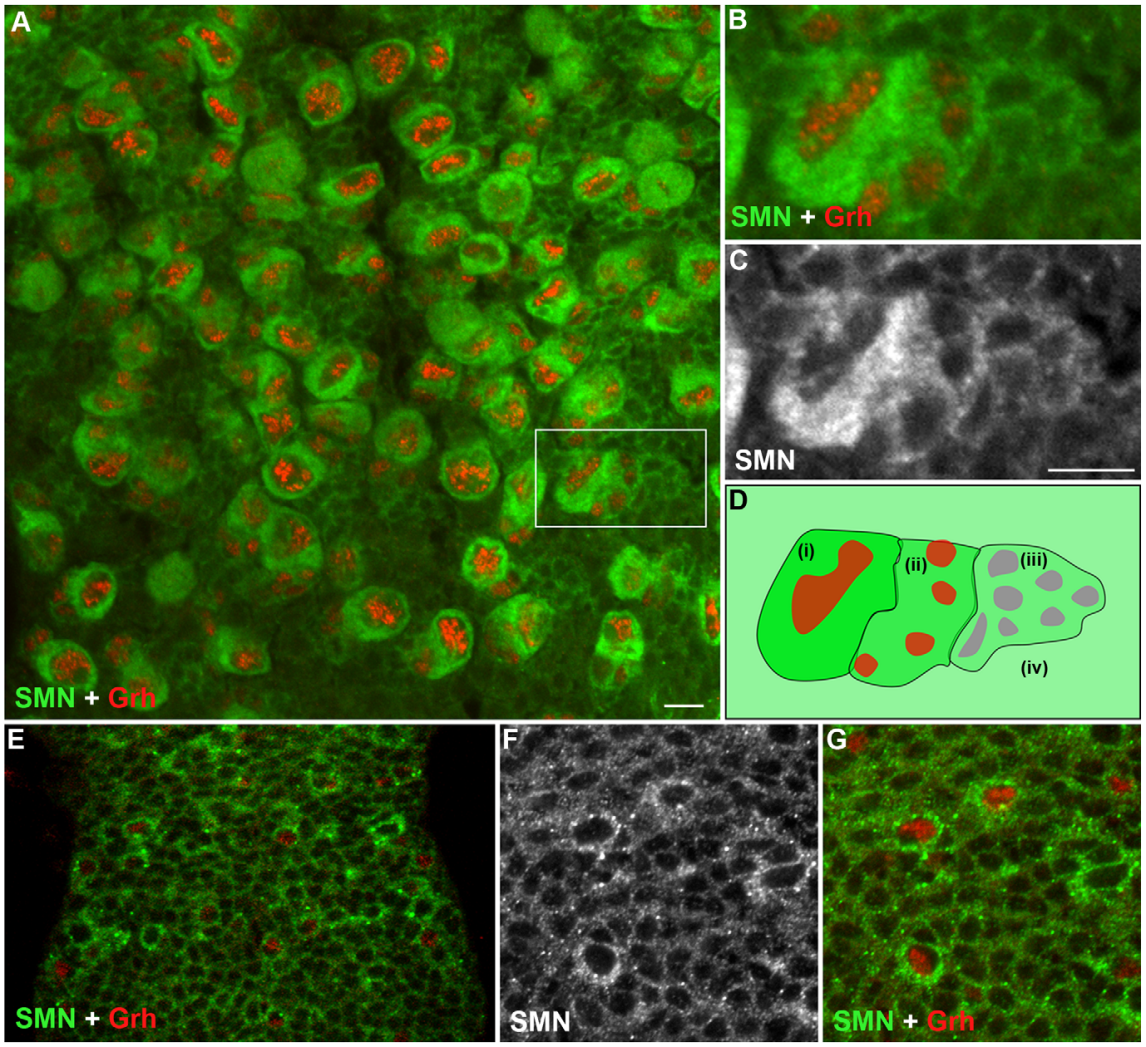
SMN is enriched in the postembryonic neuroblast (pNB) and forms a gradient in the developing neurons. (A) A section of the 3^rd^ instar thoracic ganglion showing SMN enriched in pNBs labelled with the pNB specific transcription factor Grh. The hatched box shows the pNB and daughter cells in (B) and (C) A gradient of SMN from pNBs through to the daughter cells in the same clone. (D) A schematic of the SMN gradient from (i) the most intense to (iv) the least intense. (E–G) SMN is enriched in pNBs of a late 1^st^ instar larvae. (F, G) A separate scan of the pNBs in E. The antibody used was rabbit anti-SMN (gift from Jianhua Zhou 1∶2000). Scale bars, 10 µm.

Although a ubiquitously expressed protein, SMN has been shown to be regulated during cell differentiation. To understand how SMN may control the generation of new neurons, we analysed SMN levels in the ventral ganglion of the developing larval CNS (Figure 2). In the early 1^st^ instar larval CNS, SMN staining was ubiquitous and localised in punctate bodies. During the late 1^st^ and 2^nd^ instar stages SMN levels increased in cells that correspond to the quiescent pNBs (Figure 2E–2G). SMN enrichment coincided with the expression of Grainyhead (Grh), a transcription factor and pNB marker, [18] and prior to the detection of Miranda (Figure 2). SMN accumulation increased as the pNBs enlarged with the highest expression of SMN protein found in the cytoplasm of 2^nd^ and 3^rd^ instar dividing pNBs (Figure 2A–2C; 3^rd^ instar pNBs). Each pNB divides asymmetrically producing a large cell, which retains neuroblast identity, and a smaller cell termed the ganglion mother cell (GMC), which divides terminally into two postmitotic progeny. The intensity of SMN expression in the adjacent GMC was slightly lower. Levels in the cytoplasm then decreased in a gradient through the daughter cells until it resided to a basal level in the differentiated neurons and glia. In the *Drosophila* larval brain both type I (ID and IA) and type II neuroblasts generate progeny through different intermediary precursor cells [19], [20]. We have found SMN is enriched in type ID (thoracic and brain lobe), type IA and all the Miranda staining neuroblasts of the brain lobes (Figure S1). SMN functions within a complex with a group of proteins called the Gemins. We have also found that Gemin5 has a comparable pattern in the CNS (Figure S2).

### SMN loss alters the basal localisation of Miranda

**Figure 3:**
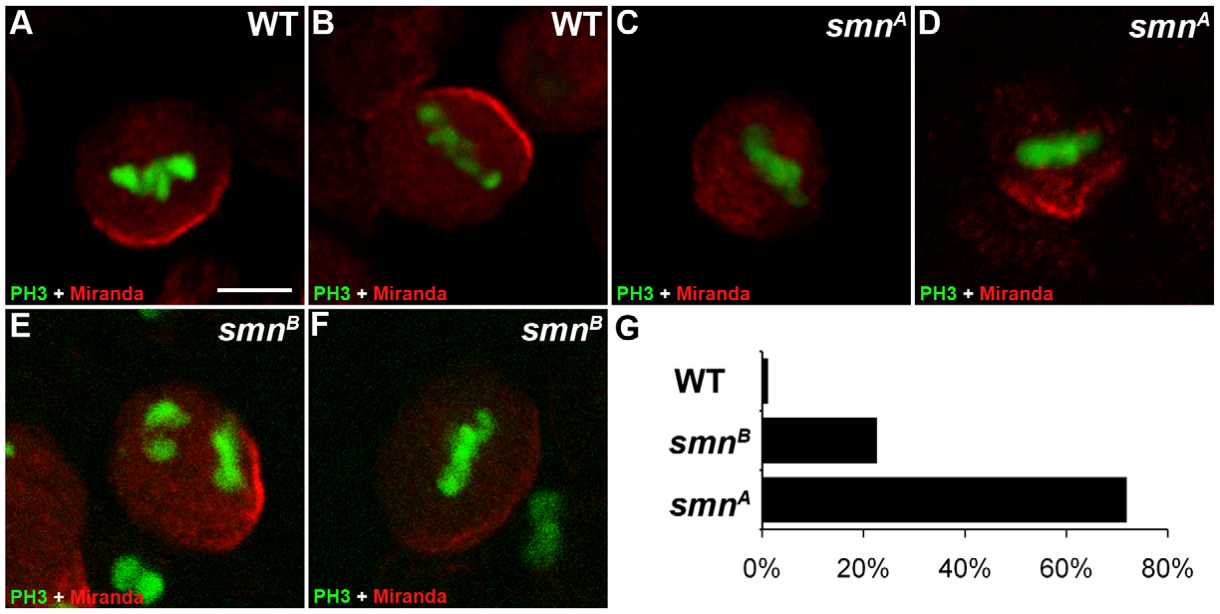
Miranda localisation is abnormal in *smn* mutant neuroblasts. Wild-type (A, B), *smn^A^* (C, D), and *smn^B^* (E, F) post embryonic neuroblasts (pNBs) showing Miranda and PH3 staining. During metaphase Miranda forms a crescent along the membrane of the neuroblast (A, B). In the *smn* mutant Miranda staining is reduced at the metaphase membrane (C and F) or can appear punctuate in the cytoplasm of the pNB (D). (G) Quantification of Miranda mislocalisation for each genotype. pNBs with inaccurate localisation, low level or the punctate localisation of Miranda were scored. Scale bar, 10 µm.

Miranda is a cargo protein that forms a messenger ribonucleoprotein (mRNP) complex with *prospero,* a transcription factor that drives daughter cell differentiation [21]. Before division, Miranda protein localises to the apical membrane of the neuroblast directing the basal localisation of *prospero* mRNA [22]. These proteins are arranged on the basal membrane during late prophase and metaphase and become segregated into the GMC upon cytokinesis. To further understand the function of SMN in pNBs, we looked to see if Miranda localisation was affected (Figure 3). In wild-type cells Miranda was asymmetrically localised as a crescent on the neuroblast membrane parallel to the metaphase chromosomes (Figure 3A, 3B). The metaphase chromosomes were labelled with phosphorylated histone H3 (pH 3). pH 3 is a marker that is expressed at M phase. Phosphorylation of histone H3 on serine-10 promotes the condensation of chromatin, an event tightly linked to the entry into mitosis. Both *smn^A^* (Figure 3C, 3D) and *smn^B^* (Figure 3E, 3F) neuroblasts displayed defective Miranda localisation. In 23% of *smn^B^* neuroblasts (n = 91) and 71% of *smn^A^* neuroblasts (n = 42), Miranda did not correctly localise in a crescent during metaphase, and could be seen to be diffuse or punctate in the cytoplasm, or in a band that was not localised correctly in relation to the metaphase chromosomes (Figure 3G).

### Mutant SMN pNBs have defective divisions

**Figure 4:**
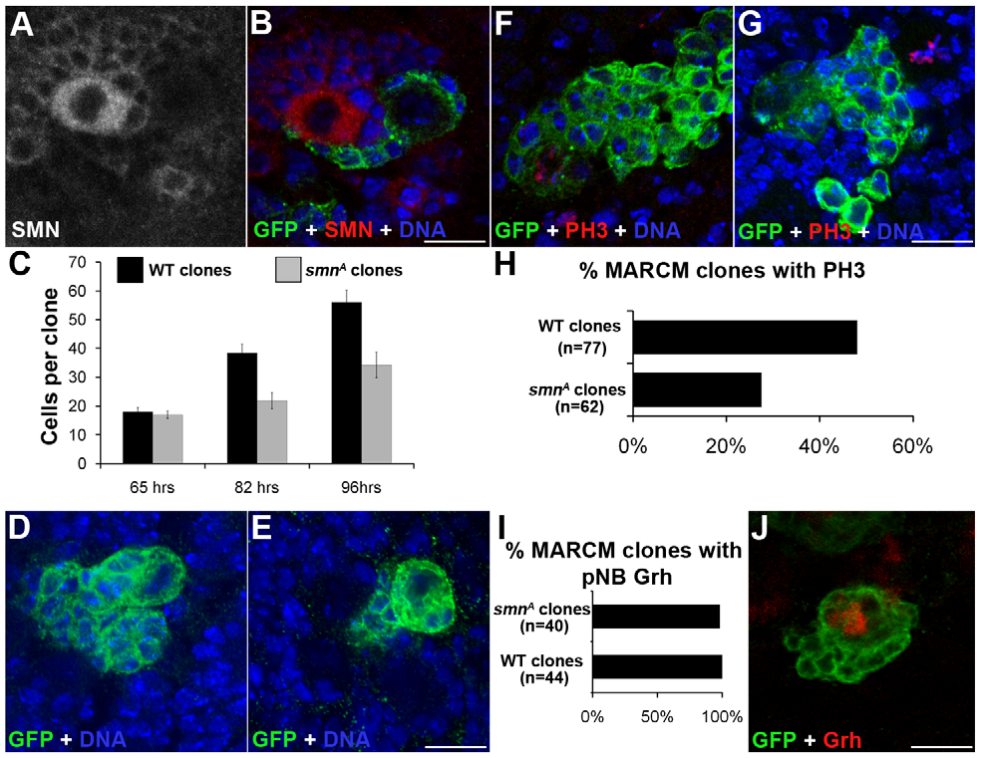
MARCM analysis of SMN in the larval CNS. (A, B) Single frame images of a *smn^A^* MARCM clone showing the reduction of SMN. (C) Quantitative analysis of cell numbers in MARCM clones. Cell number counts at 65 hrs (wild-type, µ = 18; *smn^A^*, µ = 17), 82 hrs (wild-type, µ = 38.3; *smn^A^*, µ = 21.8; P<0.005, T-Test, n = 86) and 96 hrs (wild-type µ = 56.1; *smn^A^*, µ = 34.2; P<0.001, T-Test, n = 95). *smn^A^* clones show a significant reduction of cell proliferation over time. (D, E) Confocal pictures of a 64-hr wild-type and *smn^A^* clone. *smn^A^* clones have an abnormal shape, with GMCs formed in a inconsistent pattern. Clones imaged at 64 hrs. (F, G) Wild-type and *smn^A^* mutant MARCM clones stained for PH3. (H) The number of wild-type and *smn^A^* clones with pNBs positive for pH3 were counted and displayed as a percentage of the total number of clones from each type (wild-type  = 48%, n = 54; *smn^A^*  = 24% n = 56; P<0.001, T-Test). (I, J) *smn^A^* clones were stained for Grainyhead (Grh) to detect the presence of a neuroblast. (I) The majority of *smn^A^* clones have a large Grh positive cell present. Scale bars, 10 µm.

We used the mosaic analysis with a repressible cell marker (MARCM) system to visualize pNB clones lacking SMN with the positive marker GFP [23]. When double-stained with antibodies against SMN and GFP, *smn* MARCM clones (GFP positive) had a low SMN signal (Figure 4A, 4B), in contrary to their neighbouring GFP-negative cells. In wild-type clones SMN enrichment was still present (Figure S3). The GFP clones represent pockets of immature neurons that will become the motor neurons and glial cells of the adult fly. To analyse how SMN reduction intrinsically affects the thoracic type IID larval neuroblasts, we heat-shocked wild-type and *smn^A^* MARCM crosses and analysed clones at 65-, 82- and 96-hr post hatching. Quantitative analysis shows *smn^A^* mutant pNB clones were significantly smaller than control clones derived from wild-type pNBs (Figure 4C, 4E). GMCs separated from the pNB in an inconsistent pattern generating mutant clones that were abnormally arranged (Figure 4E). Many of the *smn^A^* clones resided on the ventral surface and often daughter cells were seen adjacent to but not completely part of the pockets of cells (Figure 4B and 4E). Clones at the 96-hr stage were also stained for pH 3. The number of wild-type and *smn* clones with pNBs and GMCs positive for pH 3 were counted and displayed as a percentage of the total number of clones from each type (Figure 4F–4H). Wild-type control clones were stained with pH 3, a mitotic marker, in 48% of the clones. In contrast only 24% of *smn^A^* clones were shown to be in M phase at that time. This suggests that the *smn* mutant clones are in M phase less often and therefore dividing less than wild-type pNBs. Both *smn^A^* and *smn^B^* mutant larvae had a reduction of pH 3 in the thoracic ganglion (Figure S4).

To see if stem cells within the clones were lost we stained 96-hr old clones for the pNB marker Grh. Almost all (98%) of the *smn^A^* mutant clones contained a large Grh-positive cell suggesting stem cell loss had not occurred at this stage (Figure 4I). *smn^A^* and *smn^B^* larvae were also stained for activated caspase-3 to test for apoptosis, and Hsp70 to test for stress. In all cases these markers were not present in the pNBs or GMCs in the thoracic ganglion of the mutants, suggesting the reduction in proliferation was not due to pNB death (Figure S5).

### SMN loss in pNBs leads to reduced snRNA levels

**Figure 5:**
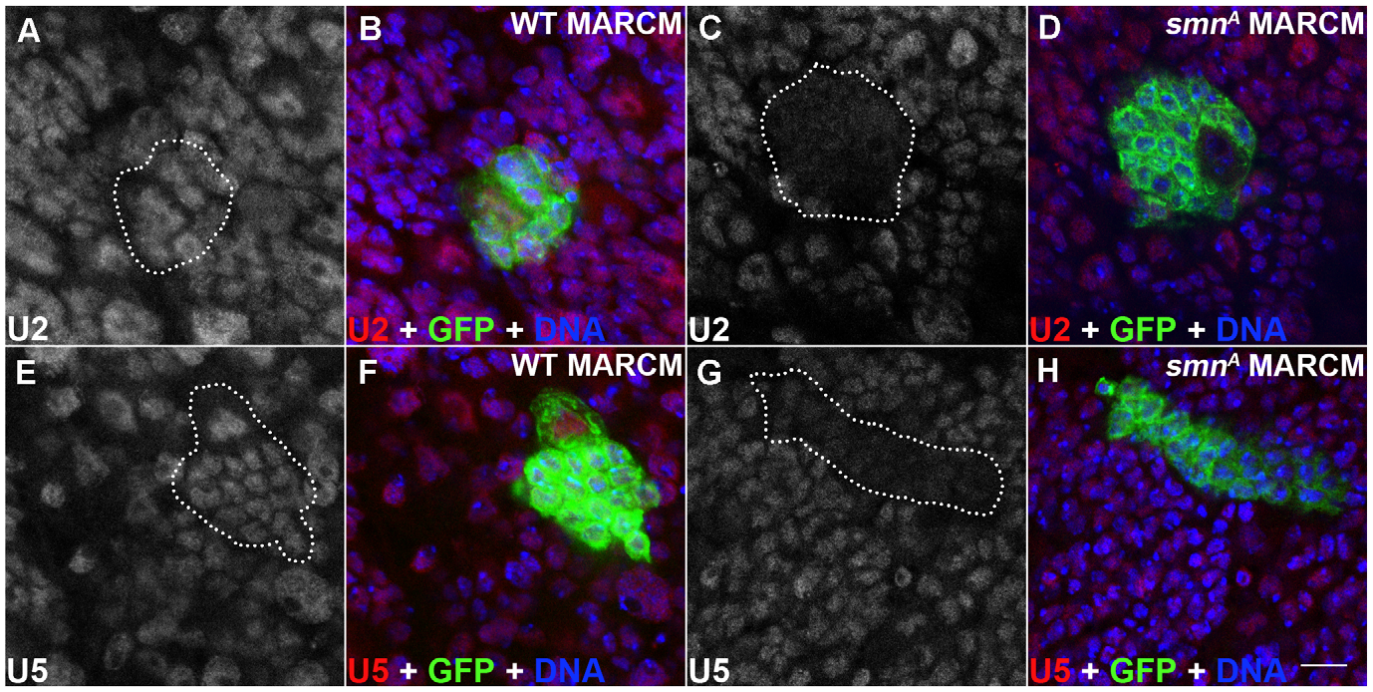
snRNP levels are reduced in SMN mutant MARCM clones. (A–H) U2 and U5 expression in wild-type and *smn^A^* pNB MARCM clones using *in situ* hybridisation. Each clone is only one segment of a z-stack. Clones are labelled with GFP. U2 and U5 expression is located in the nucleus of wild-type clones (U2: A,B; U5: E, F) and in non mutant cells. SMN levels are reduced in *smn^A^* mutant clones (U2: C, D; U5: G, H). Scale bar, 10 µm.

SMN, in complex with a set of proteins called the Gemins, promotes the assembly of uridine-rich snRNPs which are components of the spliceosome. snRNPs consist of an Sm protein ring and a number of uridine-rich snRNAs that include U2 and U5. In *Drosophila*, a minimal SMN complex consisting of SMN, Gemin2 (SIP2), Gemin3 and Gemin5 (Rig) has been reported [16], [24], [25], [26]. To understand if SMN reduction in the dividing neuroblasts affects U snRNAs, U2 and U5 levels were tested using *in situ* hybridisation in *smn^A^* mutant clones (Figure 5) [27], [28]. It was previously reported that there are no gross changes in snRNA levels in *smn^A^* and *smn^B^* mutant larvae [29]. However in this study both U2 and U5 levels were reduced in *smn^A^* mutant MARCM clones suggesting snRNAs in the developing *Drosophila* neurons may be particularly sensitive to SMN reduction (Figure 5C, 5D, 5G, 5H). U2 and U5 were observed in a consistent pattern in both the surrounding wild-type cells in the *smn^A^* MARCM clones, and in the wild-type MARCM clones (Figure 5).

### Disruption of SMN alters stem cell longevity in the testis

*Drosophila* testes offer a tractable system to study stem cell maintenance and cell differentiation. In addition, *Drosophila* testes have the highest number of alternative splicing events and exhibit prominent changes in the expression of snRNPs and splicing factors as sperm develop [30].

**Figure 6:**
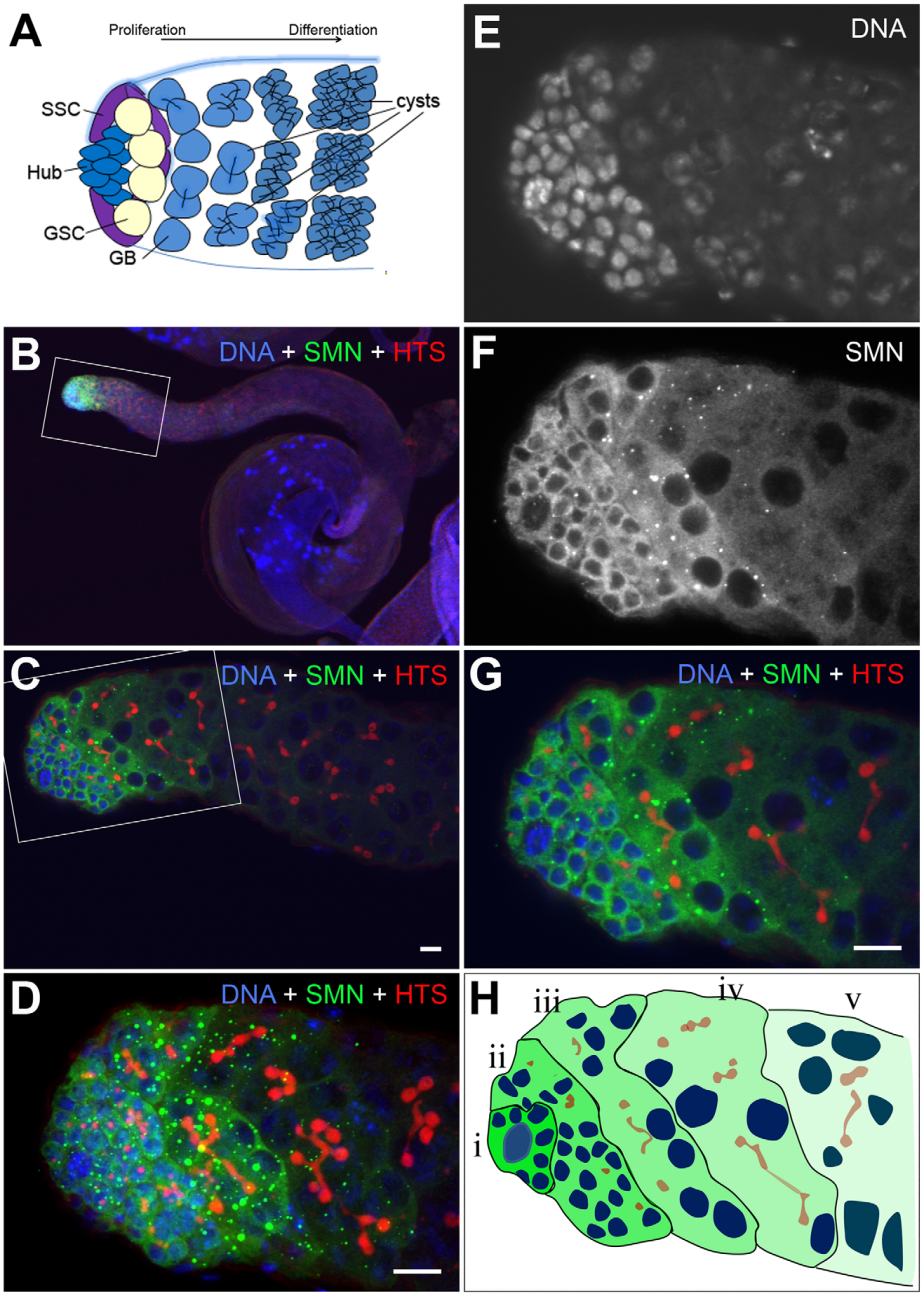
An SMN gradient in the *Drosophila* adult testis. (A) Schematic of the larval testis showing the position of germline stem cells (GSC), somatic stem cells (SSC), gonialblast (GB) and spermatogonia. (B–D) In *Drosophila* testis SMN staining is localised to the apical tip. (E–G) SMN is enriched in GSCs and levels decrease as cells differentiate. (H) Schematic showing the gradient in the testis from (i) the most intense to (v) the least intense. HTS (hu-li tai shao) labels the spectrosomes. The antibody used was rabbit anti-SMN (gift from Jianhua Zhou 1∶2000). Scale bars, 10 µm.

In addition to the larval CNS, SMN protein formed a striking gradient in *Drosophila* testes (Figure 6). In adult testes, 8–12 germline stem cells (GSCs) surround the hub cells which serve as the somatic niche. Each GSC divides into two daughter cells. One daughter cell remains as a stem cell, while another daughter cell differentiates into a gonialblast (GB). GBs divide four times to produce 2-, 4-, 8- and 16-cell cysts (Figure 6A) [31]. Each spermatogonial cell in a 16-cell cyst will undergo meiosis giving rise to 4 spermatids. SMN staining was virtually undetectable in the hub cells, whilst it was enriched in GSCs. SMN levels remained very high in GBs and spermatogonia with predominant punctate structures resembling U bodies. SMN levels then decreased dramatically in spermatocytes (Figure 6B–6G). SMN formed a clear gradient from GSCs to their differentiated progeny in adult testes (Figure 6H).

**Figure 7:**
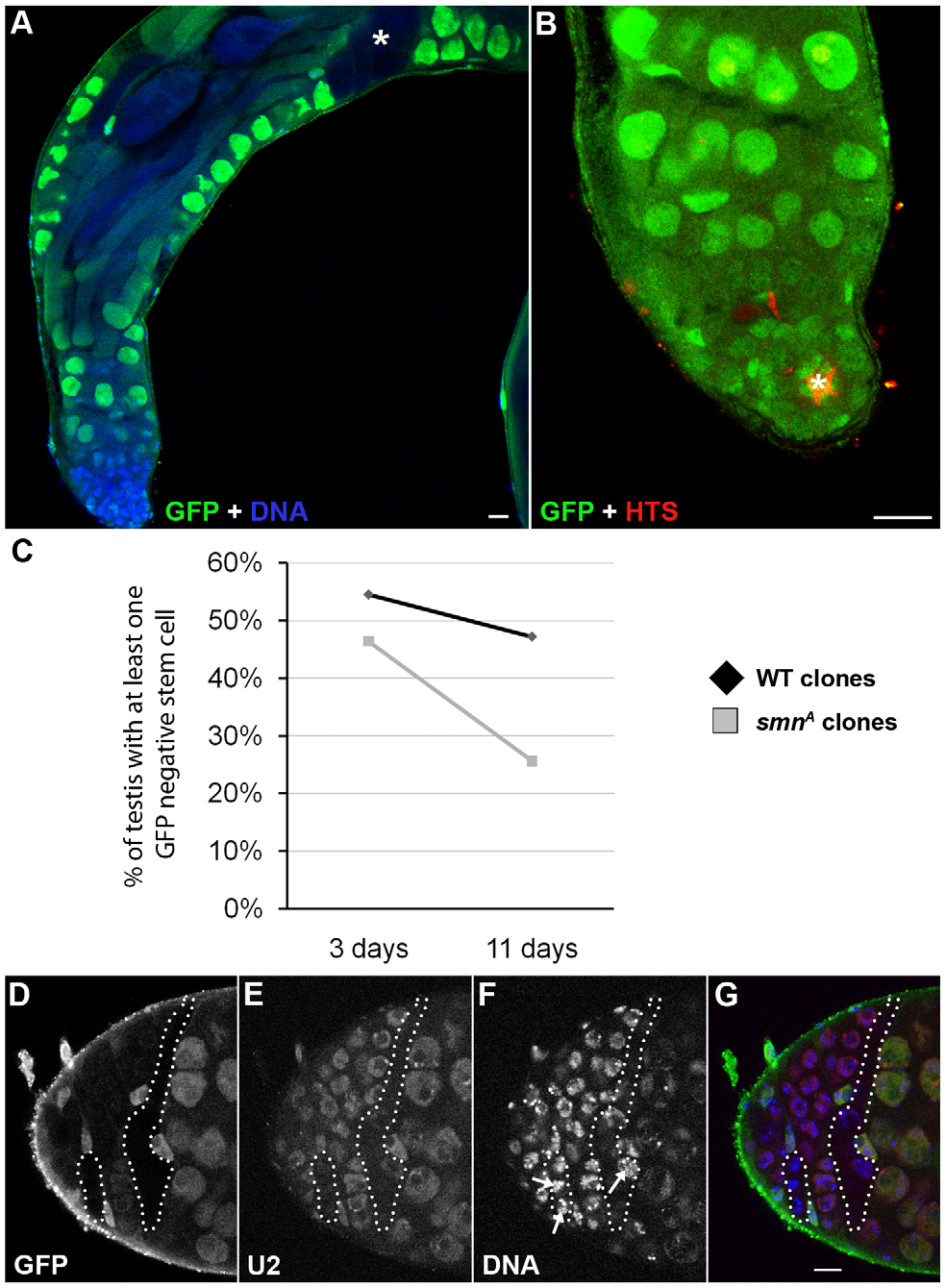
SMN is essential for the maintenance of male germline stem cells. (A, B) *smn^A^* mitotic clone in the *Drosophila* adult testis. Testes with GFP negative goniablasts and spermatocytes (A, *) but no GFP negative stem cell are scored. Stem cells are recognised by their proximity to the hub (B, *, FasIII positive). (C) Stem cells are lost at a greater rate in *smn^A^* mitotic clone testis when compared to a wild-type control. Stem cells were counted in control and *smn^A^* testis at 3 (control, n = 55; *smn^A^*, n = 56) and 11 (control, n = 72; *smn^A^*, n = 78) days. There was very significant stem cell loss in *smn^A^* at 11 days (p<0.001, T-test) when compared to the control. (D–G) U2 levels are reduced in *smn^A^* mitotic clones. The hatched region shows GFP negative clones. Arrows point to SMN negative nuclei with low U2. Scale bars, 10 µm.

Stem cell loss was also observed in testis mitotic clones which were studied over an 11-day period (Figure 7). In contrast to MARCM, *smn* mutant cells are GFP negative when using the mitotic clonal system. Clones that contained GFP-negative gonialblasts and primary spermatocytes, but no GFP-negative GSCs, were regarded as having lost the GSCs that would have previously generated the GFP negative cells observed (Figure 7A, 7B). This enables the identification of clones where the stem cell is lost. The number of *smn^A^* mitotic clones that lacked a GFP-negative stem cell was considerably higher than the wild-type clones in this study. This difference became more pronounced at day 11 indicating SMN is essential for the survival of GSCs in testes. Quantitative analysis showed that the longevity of male GSCs generating *smn^A^* clones was greatly reduced (Figure 7C). To test whether snRNP levels were also altered in GSC clones, we analysed the mitotic clones with a combination of immunostaining and fluorescence *in situ* hybridisation. In *smn^A^* mutant clones U2 levels were reduced (Figure 7D–7G). These results suggest that SMN is required for both the proliferation and survival of GSCs, and the maintenance of snRNA levels in stem cell clones.

### SMN overexpression alters tissue growth and pupation timing

**Figure 8:**
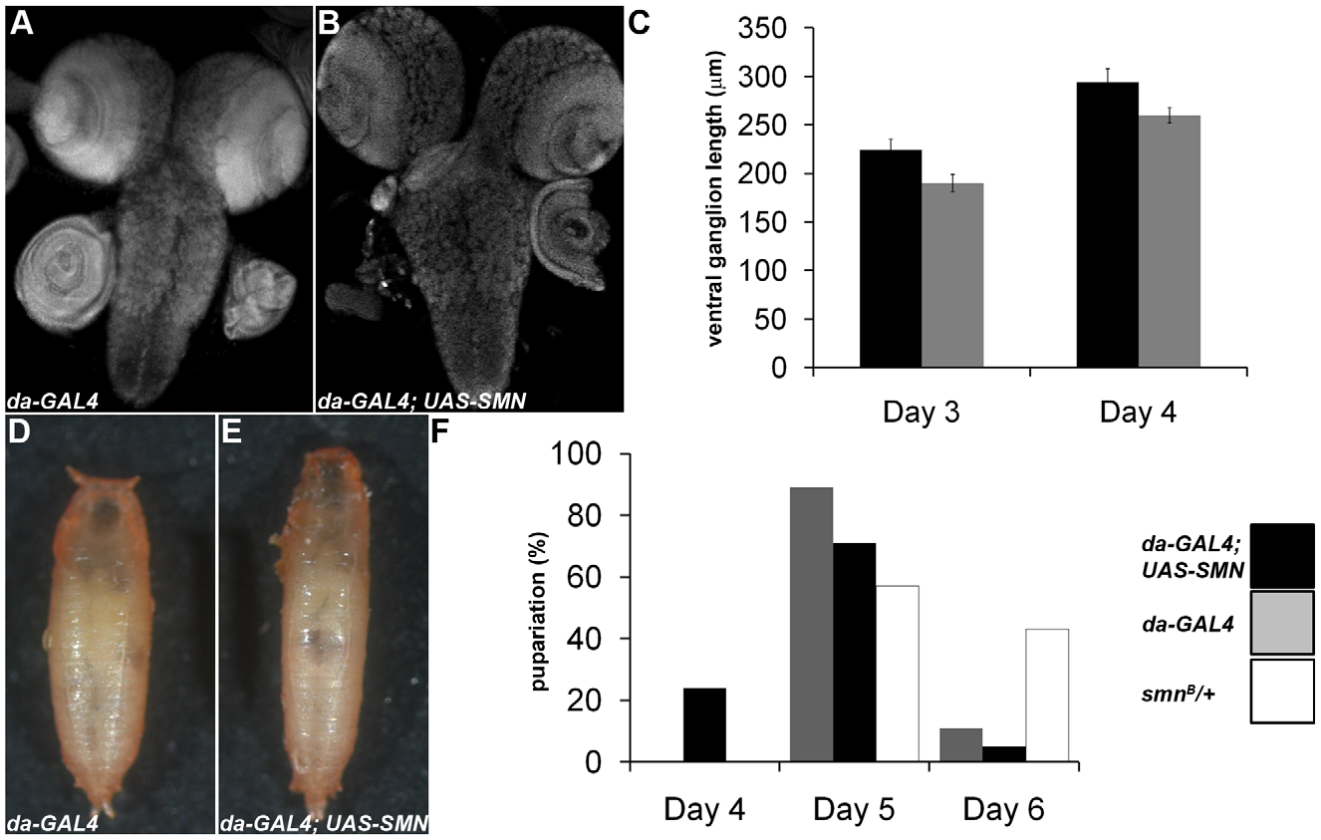
SMN overexpression accelerates CNS development and pupation entry. *da-GAL4* and *da-GAL4*; SMN-YFP larvae were analysed to understand how SMN up-regulation affects CNS development and pupation. (A–C) At 3 and 4 days after hatching the ventral nerve cord of the larvae were measured. *da-GAL4*; SMN-YFP CNS are larger at each time point. (F) A significant proportion of the da*-GAL4*; SMN-YFP larvae enter pupation early when compared to the *da-GAL4* controls. The prematurely pupating *da-GAL4*; SMN-YFP pupal cases fail to contract fully and appear elongated (D, E).

Our results have shown that SMN enrichment in larval neuroblasts and male GSCs is required for proper proliferation, snRNA levels and Miranda localisation. In addition, SMN levels are strongly downregulated upon differentiation. Using *daughterless-GAL4* (*da*-GAL4) and a functional SMN-YFP transgene, we looked to see how ubiquitous overexpression of SMN affects development. Flies with transgenic expression of SMN using *da-GAL4* were viable but exhibited numerous growth defects. To understand how SMN overexpression affects larval CNS growth we measured the length of the ventral ganglion along the ventral midline (Figure 8). Embryos from each genotype were collected over a 1-hr laying period and grown at 25°C with comparable food amounts and population densities. The CNS from *da-GAL4*; SMN-YFP larvae increased in size quicker than that from wild-type animals (Figure 8A, 8B). Upon pupation the pupae cases of *da-GAL4*; SMN-YFP also failed to contract fully and appeared elongated (Figure 8D, 8E). However, these pupae were viable. In addition, *da-GAL4*; SMN-YFP larvae had irregular pupation patterns with many larvae pupated earlier than the *da-GAL4* controls (Figure 8F).

### SMN overexpression alters the timing of cell differentiation in testis

**Figure 9:**
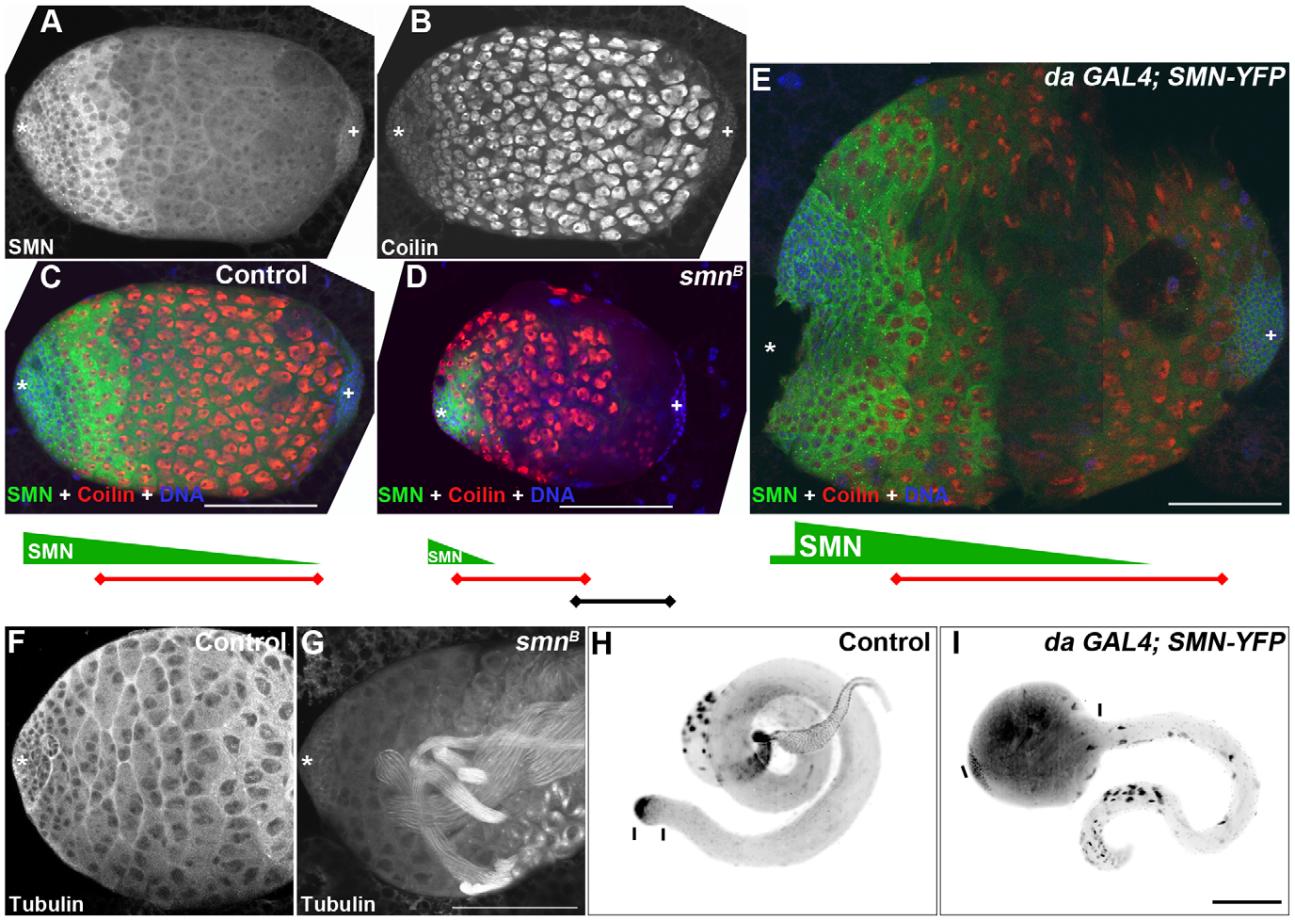
Disruption of the SMN gradient affects differentiation timing in the testis. (A, B) 3rd instar larval testis showing the SMN gradient and coilin staining in the spermatocytes. (C, D) Testis from control (C), *smn^B^* mutant (D) and *da-GAL4*; SMN-YFP (E) larvae. Green bars represent the SMN gradient; red bars represents the band of coilin stained spermatocytes; star, apical stem cells; plus, distal terminal cells. (D) *smn^B^* mutant testis. SMN gradient is limited to the apical end cells and the spermatocyte band move apically. (E) Testis from a larva overexpressing (*da*-GAL4) SMN. SMN gradient extends further to the terminal end and the apical boundary of the spermatocyte region moves towards the terminal end in *da-GAL4*; SMN-YFP testis. Note the size differences in (C, D, and E) which are to the same scale. Wild-type (F) and *smn^B^* (G) 4-day old 3rd instar larval testis stained for α tubulin. *smn^B^* mutant testis regularly (85%, n = 28) have multiple sperm tails present. These mature sperm appear closer to the apical hub and germline stem cells (star). (I) Overexpression of SMN suppresses cyst cell differentiation and induces ‘tumour’ formation in the spermatogonial region. Note the distances between the bars in H and I which show the proportion of cyst cells in the testis. Scale bars, 100 µm.

We next wanted to understand how altering SMN levels can affect the downstream progeny of stem cells. We used *Drosophila* larval testis as a model since the SMN expression gradient is the most striking in this system (Figure 9). The 3^rd^ instar larval testis is an oval-shaped organ with bands of distinct cell types residing along the apical-terminal axis. GSCs, GBs and cysts occupy the apical fifth, while terminal cells occupy the terminal fifth. The central three fifths of the testis consist predominantly of large spermatocytes which can be identified with bright coilin staining (Figure 9B) [32]. This pattern is consistently observed in wild-type larvae and is compatible with the correct temporal development of mature sperm for the adult fly. Consistent with adult testes, SMN formed a gradient in larval testes with high levels of SMN in GSCs, GBs and decreased levels in cysts and spermatocytes (Figure 9A). As developing cells migrate further away from the apical stem cells, the level of SMN protein decreased.

Based on the striking inverse correlation of SMN concentration and cell differentiation, we hypothesised that SMN could affect the differentiation of specific cell types. To test this idea, we examined larval testes with overexpressed and low levels of SMN (Figure 9C–9E). Embryos were collected over a 2-hr laying period and testes were dissected 4 days after hatching. In 3^rd^ instar *smn^B^* testis, residual amounts of SMN were present due to low levels of maternal contribution (Figure 9D). An SMN gradient was still present in *smn^B^* testes but was restricted to the apical tip. Levels then sharply declined until undetectable in cells distal to the apical end. Consistent with the shift of the SMN gradient, the terminal side of the spermatocyte band retracted to the apical terminus causing an increase in the number of differentiated cells. Mature sperm were present at this stage within this region (Figure 9F, 9G). Groups of cells in this region also underwent apoptosis. Overall the mutant testes were smaller than same-stage wild-type controls.

In contrast, overexpressing SMN-YFP that was driven by *da-GAL4* induced a migration of the SMN gradient towards the terminal end (Figure 9E). *da-GAL4*; SMN-YFP was expressed in the whole testis, however the most intense expression was in the primary spermatocye band. Consequently, SMN levels appeared very low around the hub and stem cells (Figure 9E). This high expression caused the apical boundary of the spermatocyte region moves towards the terminal end. The size of *da-GAL4;* SMN-YFP testes could be at least 3 times larger than testes from wild-type larvae. To understand this phenotype further the morphology and development of sperm was analysed in adult testes. Similarly, overexpression of SMN caused an increased number of primary spermatocytes in adults, creating an enlarged tumour-like phenotype (Figure 9H, 9I). In addition very few mature sperm were present in the adult suggesting that ectopic expression of SMN represses the differentiation of spermatogonia into sperm.

## Discussion

This study shows a high demand for SMN in *Drosophila* stem cells. In addition, we have identified a striking SMN concentration gradient, inversely proportional to the state of differentiation, in *Drosophila* larval CNS and testis. In *Drosophila* SMN mutant larvae, both the CNS and testis display growth defects which precede the previously reported motor defects and death. These larvae also fail to localise Miranda protein correctly at the basal membrane of the neuroblast. Clonal analysis indicates that SMN deficient stem cells have a reduced number of divisions and also generate cells with lower levels of U2 and U5 snRNPs. Overexpression of SMN alters the timing of CNS growth and disrupts the onset of pupariation and pupation. Using the male germline system, we show that prolonged SMN reduction leads to stem cell loss. Finally we find that ectopic SMN expression in cells along the SMN gradient leads to changes in the timing of cell differentiation. We therefore suggest that the fine-tuning of SMN levels throughout development can lead to complex developmental defects and reduce the capacity of stem cells to generate new cells in development.

SMN levels have been reported to be extremely high in early development [11]. We show that SMN up-regulation occurs in neuroblasts prior to the initiation of their cell division, suggesting a distinct increase of SMN levels is required for new rounds of neurogenesis and local proliferation. Fewer immature neurons are generated in the thoracic ganglion of *smn* mutant MARCM clones. Provisional data has suggested there may be proliferation defects in the spinal cord of severe mouse models [33]. In addition, a recent study using the severe SMA mouse model has shown proliferation defects in the mouse hippocampus, a region associated with higher SMN levels [34]. Together these data suggest that, in part, the pathology observed in more severe forms of SMA may be caused by defects in tissue growth.

Proteins involved in processes such as chromatin remodelling, histone generation and cell signalling have been identified as intrinsic factors for the maintenance of *Drosophila* stem cells. To the best of our knowledge, this is the first report of stem cell defects caused by the reduction of a protein involved in snRNP biogenesis. Although SMN is required in all cells, proper stem cell function requires a substantially higher level of SMN. This study also shows snRNP defects in *Drosophila* SMN mutant tissue. Previous studies in *Drosophila* have shown no gross changes in snRNP levels, including U2 and U5, in lysates from whole *smn^A^* and *smn^B^* mutant larvae [29]. *smn^A^* MARCM neuroblast clones and male germline mitotic clones have reduced snRNP levels, suggesting snRNP assembly may be particularly sensitive to SMN reduction during CNS and germline development.

SMN mutant neuroblasts have abnormal Miranda localisation. Miranda, an adaptor protein, forms a complex with the RNA binding protein Staufen which binds to *prospero* mRNA [35], [36]. In addition to snRNPs, SMN protein has been implicated in the biogenesis of numerous RNP subclasses, including proteins involved in the transport and localisation of β-actin mRNA at the synapse. Whether Miranda mislocalisation is due to direct or indirect associations with SMN should be addressed.

SMN mutant larvae have been previously shown to have synaptic defects which include enlarged and fewer boutons and a reduction in the number of GluR-IIA clusters – the neurotransmitter receptor at the *Drosophila* neuromuscular junction [12], [24]. In addition, numerous developmental defects are observed including pupation and growth defects. Complementing this work, *Drosophila* Gemin5 a member of the *Drosophila* SMN-Gemin complex has been shown to interact with members of the ecdysone signalling pathway responsible for initiating pupation and growth [37]. *Drosophila* Gemin5 is also enriched in pNBs, in a pattern comparable to SMN (Figure S5). There is increasing evidence that suggests the *Drosophila* SMN complex plays an important role in pupation. Ubiquitous overexpression of SMN using *da-GAL4* advances CNS development and causes premature entry into pupation. The ecdysone pathway has been identified to play an important part in the regulation of neuroblast division and neuronal differentiation during development [38], [39]. How the *Drosophila* SMN complex plays a part in stem cell biology, and how the SMN complex interacts with specific signalling pathways should be the subject of further study.

Larval and adult testes exhibit the most distinct SMN gradients in *Drosophila* tissues. *Drosophila* testes have a constant population of germline stem cells that start to divide in the late larval stages and produce sperm throughout life. The removal of SMN from male germline stem cells results in stem cell loss. In the *smn^B^* mutant testis, the reduction of SMN causes a contraction of the SMN gradient towards the apical stem cells. As SMN is lost from the primary spermatoctyes, more mature sperm are observed. Increasing SMN levels leads to an increase in primary spermatocytes and a reduction in mature sperm in the adult. This result is the first to demonstrate that high SMN levels in undifferentiated cells can repress differentiation in sperm development. Interestingly, along with the CNS, *Drosophila* testes have the highest number of alternative splicing events and the most differentially expressed splicing factors during development [30]. Understanding if differential expression of SMN in specific cell types controls a shift in splicing factors as cells switch from proliferation to differentiation will be the target of future study. A recent study has identified defects in gametogenesis and testis growth in mice lacking the Cajal body marker coilin, a binding partner of SMN [40]. The authors speculated that coilin may facilitate the fidelity and timing of RNP assembly in the cell and coilin loss may limit rapid and dynamic RNA processing. It will be important to understand how SMN and coilin genetically interact in stem cells and developing tissues.

The *Drosophila* CNS and male germline offer two new tractable systems that can be used to study SMN biology in development and stem cells. It also offers a system to study how SMN, a protein associated with neuronal development, could cause SMA. Although SMA is classically a disease of the motor neuron, a severe reduction of SMN protein affects a wide spectrum of cells including stem cells. Consistent with this idea, symptoms in mild forms of SMA (type III or IV)[41] are predominately limited to motor neurons. However, patients with the most severe type (type I), suffer from defects in multiple tissues including congenital heart defects, multiple contractures, bone fractures, respiratory insufficiency, or sensory neuronopathy [42], [43], [44], [45], [46]. Elucidating the differential requirements of SMN in individual cell types, and how their sensitivity to SMN loss can mediate the disease, can contribute to the understanding of the selectivity of SMA.

## Materials and Methods

### *Drosophila* stocks and genetics

*smn^A^* (smn73Ao) and *smn^B^*, two independently generated *smn* mutant alleles were used in this study [12], [47]. Both contain point mutations in a highly conserved carboxy-terminal domain of the SMN protein which harbours a self dimerization YXXG motif [12]. A stock with an extra copy of SMN (SMN-YFP driven by *daughterles*s-GAL4) was used for SMN overexpression experiment [28]. The transgene SMN-YFP is functional as it could rescue *smn* mutation [29]. *y w, OrgR or da-GAL4* was used as the control strains.

### Mosaic analysis with a repressible cell marker (MARCM)

To generate MARCM clones, hsFLP; *smn^A^ FRT2A/TM6b* or control *hsFLP; + FRT2A/TM6b* females were crossed to males from the MARCM driver stock *C155 ELAV-GAL4*, *UAS mCD8::GFP; P{TubP-Gal80}*, *FRT2A/TM6B* [48]. Embryos were collected over 2 hrs periods and then heat shocked at 18 to 24 hrs after laying at 37°C for 1 hr. Larvae were dissected at 65, 82, or 96 hrs for analysis.

### Generating mutant germline stem cell clones

*smn^A^* and wild-type control mitotic clones were generated using FLP-mediated mitotic recombination in adult testes [49]. *smn^A^* stem cells and progeny in mitotic clones are GFP negative. To generate stocks for clonal analysis, *Ubiquitous-GFP/TM3*, *Ser* flies were crossed to *w hsFLP*; *smn^A^ FRT2A/TM3*, *Ser* and *w hsFLP*; *+ FRT2A/TM3*, *Ser* flies. One-day-old non-Ser flies were selected for heat shocking at 37°C for two 1-hr heat shocks. Flies were fed on fresh wet yeast every day. Testes were analysed at 3 or 11 days. Stem cell maintenance and relative division rate calculations were determined as previously described by Xie and Spradling [50].

### Fluorescence microscopy

Tissue was dissected in Grace's Insect Medium, fixed in 4% paraformaldehyde in PBS and washed in 1× PBS+0.3% (v/v) Triton X-100, 0.5% normal goat or horse serum (PBT). To analyse the SMN gradient, larval CNS, adult testes were subjected to overnight staining at room temperature using three rabbit anti-SMN antibodies (Marcel van Den Heuvel, 1∶500; Spyros Artavanis-Tsakonas, 1∶2000; and Jianhua Zhou 1∶2000)[12], [51] and one mouse anti–SMN (Spyros Artavanis-Tsakonas, 1∶500)[51]. MARCM and mitotic clones were analysed using mouse anti-GFP (Abcam, 1∶500) and rabbit anti-GFP (Roche, 1∶500). Rabbit anti-Miranda (C. Doe, 1∶200), mouse anti-Grainyhead (S. Bray, 1∶5), mouse anti-HTS (1∶20), hsp70 (1∶100, Santa Cruz), activate caspase-3(1 200 Abcam), pH 3 (1∶200, Upstate Biotechnology) were also used. Alexa-Fluor conjugated secondary goat antibodies were used at a 1∶250 to 1∶1000 concentration. Samples were counterstained with nuclear-staining Hoechst 33342 (1∶500) prior to viewing with a Zeiss LSM 510 META confocal microscope. Images were processed using Adobe Illustrator. All figures in this paper were generated using rabbit anti SMN (1∶2000; gift from Jianhua Zhou).

### *In situ* hybridization

The *in situ* protocol and probes have been previously described [27], [28]. Tissue was dissected in Grace's medium and fixed in 4% paraformaldehyde in 1XPBS for detecting RNA, for 10 minutes at room temperature. Tissue is then washed in by 100 µl *in situ* mix (Formamide 20XSSC Heparin (5 mg/ml), yeast tRNA (50 mg/ml), Citric acid (0.5 M pH 6.0) DEPC H_2_O, 20% TWEEN 20) for 5 minutes. Fluorescent probes were then added and incubated for 1 hr at 37°C.

## Supporting Information

Figure S1 SMN staining throughout the larval 3^rd^ instar CNS. (A) A confocal image of the ventral ganglion and (B) the brain lobes of the 3^rd^ instar larval CNS. SMN is enriched in all Miranda positive neuroblasts. Type IA and ID neuroblasts are labelled in (A). The antibody used was rabbit anti-SMN (gift from Jianhua Zhou 1∶2000). Scale bar, 20 µm.
(TIF)

Figure S2 Gemin 5 localisation in the adult testis and larval CNS. (A) Schematic of the larval testis showing the position of germline stem cells (GSC), somatic stem cells (SSC) and spermatogonia. (B, C) dGemin5 forms a concentration gradient in the *Drosophila* testis. (D) Schematic of the larval 3^rd^ instar CNS showing the thoracic and abdominal ganglion. (D, E) dGemin5 is highly expressed in post embryonic neuroblasts in the thoracic ganglion. dGemin5 levels are lower in the abdominal ganglion, the region that contains the mature motor neurons. A, anterior; P, posterior. Scale bar, 10 µm.
(TIF)

Figure S3 pH 3 staining in the thoracic ganglion of *smn* mutant larvae. (A–C) Confocal images of wild-type (A), *smn^B^* (B) and *smn^A^* (C) CNS. The number of mitotic pNBs (pH 3) decreases in the *smn^B^* and *smn^A^* mutants. Miranda staining also fails to localise properly in the *smn* mutants. This is particularly severe in *smn^A^* (C). Scale bar, 10 µm.
(TIF)

Figure S4 Clonal analysis of SMN. (A–C) SMN straining in wild-type MARCM clones showing the SMN gradient. The antibody used was rabbit anti-SMN (gift from Jianhua Zhou 1∶2000). Scale bar, 10 µm.
(TIF)

Figure S5 Hsp70 and activated caspase-3 levels in *smn^A^* mutants. (A) Positive control for activated caspase-3 as a marker. (B, C) *smn^A^* thoracic ganglion stained for Miranda to show pNBs. Both capsase-3 and Hsp70 signals are undetectable in pNbs. Some hsp70 is observed in differentiated cells in the abdominal ganglion (arrow). Scale bar, 10 µm.
(TIF)
