# Supplementary figures and images for: Survival Motor Neuron Protein Regulates Stem Cell Division, Proliferation, and Differentiation in Drosophila
Source: PLoS Genet. 2011 Apr 7;7(4):e1002030. doi: 10.1371/journal.pgen.1002030 (PMC3072375; doi:10.1371/journal.pgen.1002030)

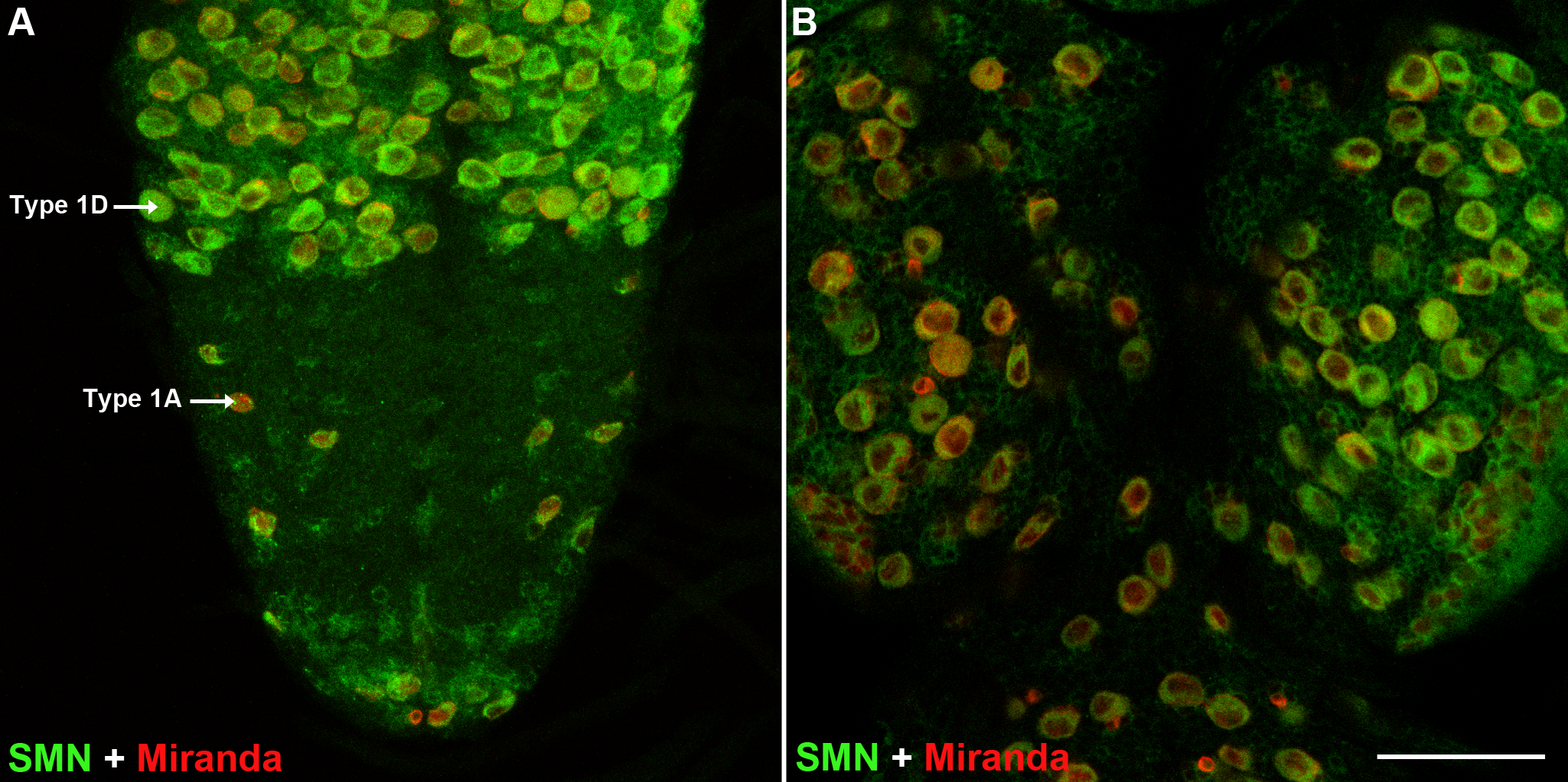

Supplement: Figure S1 — SMN staining throughout the larval 3rd instar CNS. (A) A confocal image of the ventral ganglion and (B) the brain lobes of the 3rd instar larval CNS. SMN is enriched in all Miranda positive neuroblasts. Type IA and ID neuroblasts are labelled in (A). The antibody used was rabbit anti-SMN (gift from Jianhua Zhou 1∶2000). Scale bar, 20 µm. (TIF) [file pgen.1002030.s001.tif]

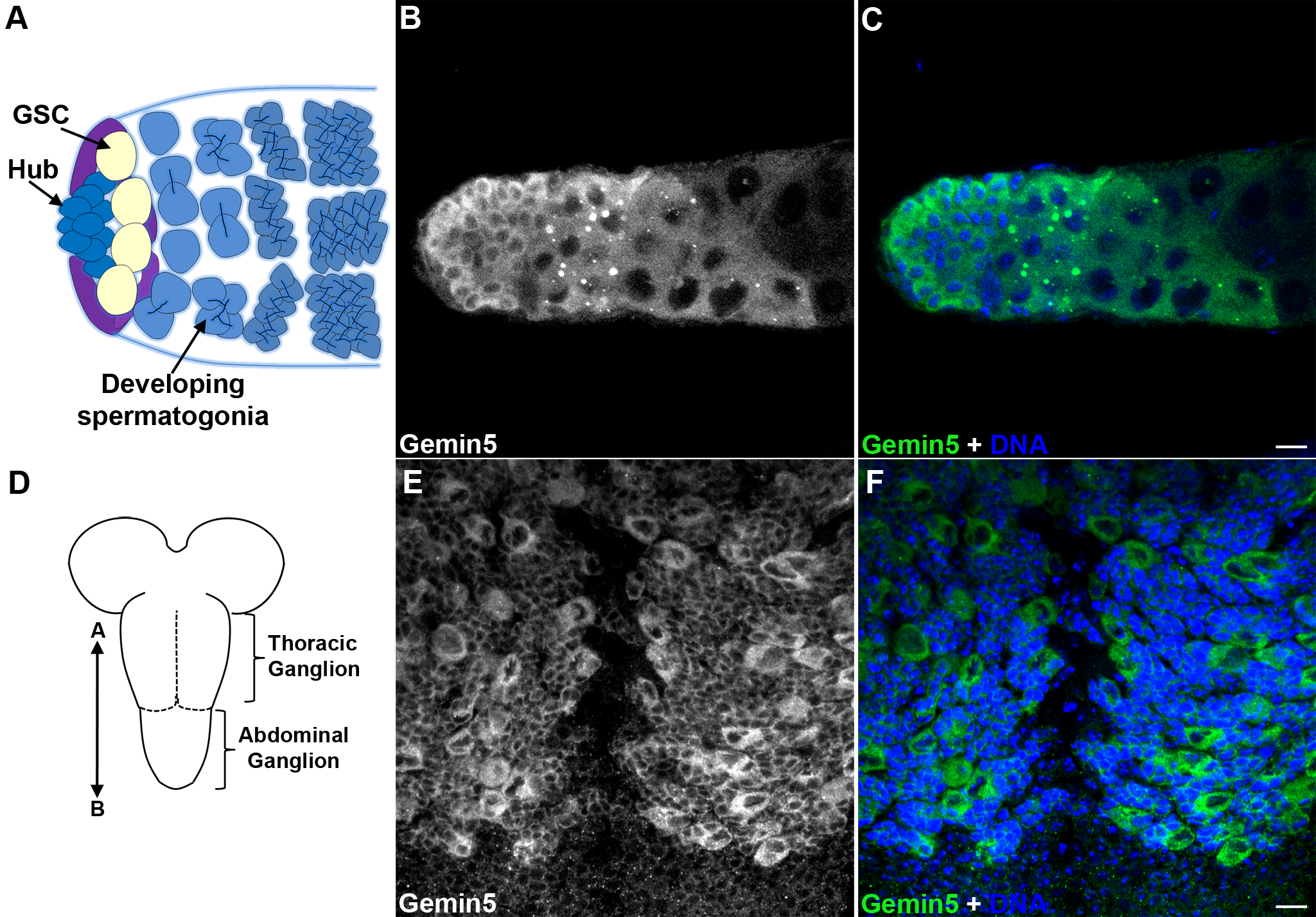

Supplement: Figure S2 — Gemin 5 localisation in the adult testis and larval CNS. (A) Schematic of the larval testis showing the position of germline stem cells (GSC), somatic stem cells (SSC) and spermatogonia. (B, C) dGemin5 forms a concentration gradient in the Drosophila testis. (D) Schematic of the larval 3rd instar CNS showing the thoracic and abdominal ganglion. (D, E) dGemin5 is highly expressed in post embryonic neuroblasts in the thoracic ganglion. dGemin5 levels are lower in the abdominal ganglion, the region that contains the mature motor neurons. A, anterior; P, posterior. Scale bar, 10 µm. (TIF) [file pgen.1002030.s002.tif]

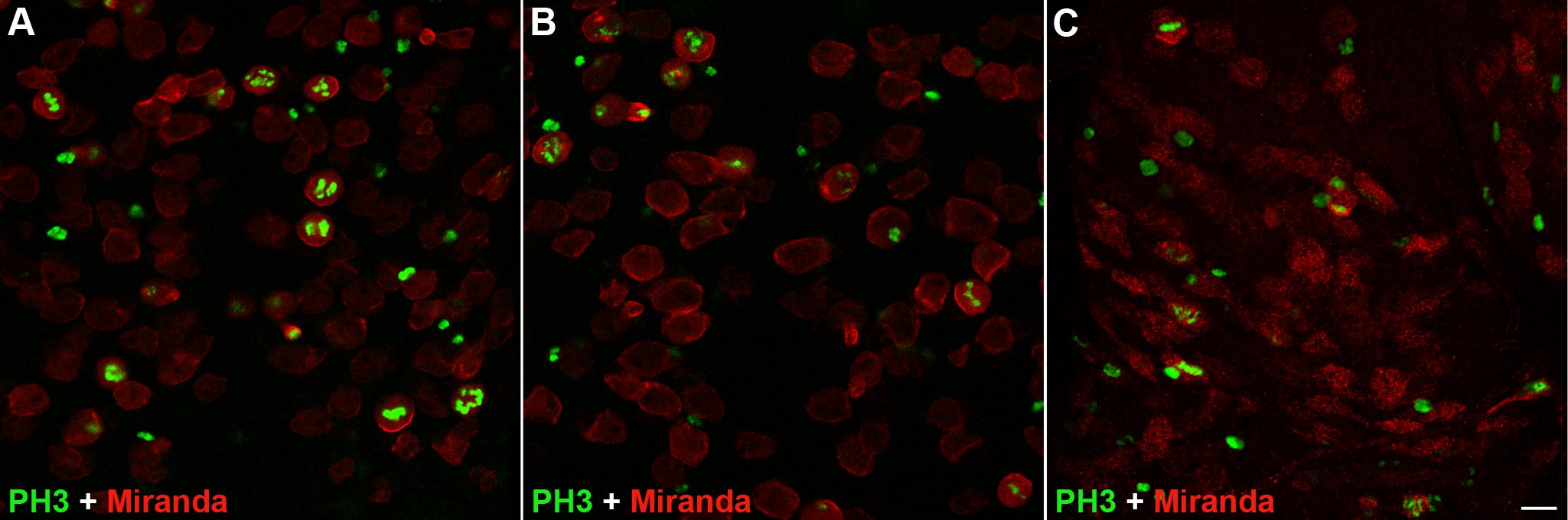

Supplement: Figure S3 — pH 3 staining in the thoracic ganglion of smn mutant larvae. (A–C) Confocal images of wild-type (A), smnB (B) and smnA (C) CNS. The number of mitotic pNBs (pH 3) decreases in the smnB and smnA mutants. Miranda staining also fails to localise properly in the smn mutants. This is particularly severe in smnA (C). Scale bar, 10 µm. (TIF) [file pgen.1002030.s003.tif]

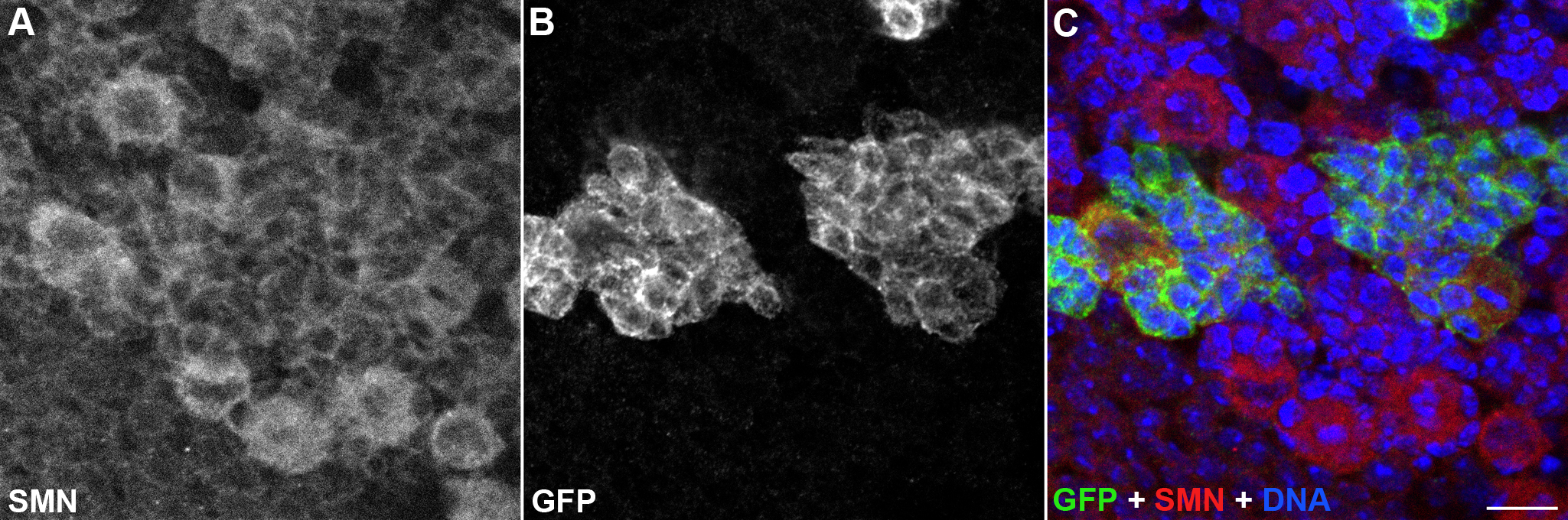

Supplement: Figure S4 — Clonal analysis of SMN. (A–C) SMN straining in wild-type MARCM clones showing the SMN gradient. The antibody used was rabbit anti-SMN (gift from Jianhua Zhou 1∶2000). Scale bar, 10 µm. (TIF) [file pgen.1002030.s004.tif]

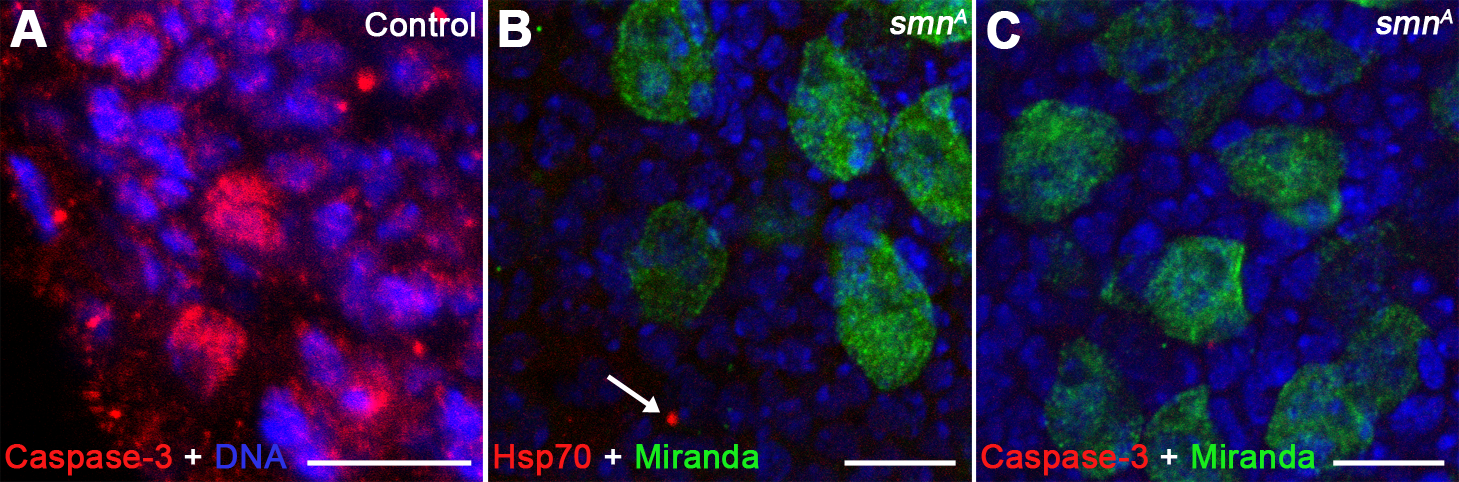

Supplement: Figure S5 — Hsp70 and activated caspase-3 levels in smnA mutants. (A) Positive control for activated caspase-3 as a marker. (B, C) smnA thoracic ganglion stained for Miranda to show pNBs. Both capsase-3 and Hsp70 signals are undetectable in pNbs. Some hsp70 is observed in differentiated cells in the abdominal ganglion (arrow). Scale bar, 10 µm. (TIF) [file pgen.1002030.s005.tif]
